# A rational approach on the use of extracorporeal membrane oxygenation in severe hypoxemia: advanced technology is not a panacea

**DOI:** 10.1186/s13613-021-00897-3

**Published:** 2021-07-12

**Authors:** Evangelia Akoumianaki, Annemijn Jonkman, Michael C. Sklar, Dimitris Georgopoulos, Laurent Brochard

**Affiliations:** 1 Department of Intensive Care Medicine, University Hospital of Heraklion, Medical School, University of Crete, Heraklion, Greece; 2 Keenan Research Centre, Li Ka Shing Knowledge Institute, St. Michael’s Hospital, Unity Health Toronto, Toronto, ON Canada; 3 Interdepartmental Division of Critical Care Medicine, University of Toronto, Toronto, ON Canada

**Keywords:** Extracorporeal membrane oxygenation, ARDS, Severe hypoxemia, Lung protective ventilation, VILI, Prone position

## Abstract

Veno-venous extracorporeal membrane oxygenation (ECMO) is a helpful intervention in patients with severe refractory hypoxemia either because mechanical ventilation cannot ensure adequate oxygenation or because lung protective ventilation is not feasible. Since ECMO is a highly invasive procedure with several, potentially devastating complications and its implementation is complex and expensive, simpler and less invasive therapeutic options should be first exploited. Low tidal volume and driving pressure ventilation, prone position, neuromuscular blocking agents and individualized ventilation based on transpulmonary pressure measurements have been demonstrated to successfully treat the vast majority of mechanically ventilated patients with severe hypoxemia. Veno-venous ECMO has a place in the small portion of severely hypoxemic patients in whom these strategies fail. A combined analysis of recent ARDS trials revealed that ECMO was used in only 2.15% of patients (*n* = 145/6736). Nevertheless, ECMO use has sharply increased in the last decade, raising questions regarding its thoughtful use. Such a policy could be harmful both for patients as well as for the ECMO technique itself. This narrative review attempts to describe together the practical approaches that can be offered to the sickest patients before going to ECMO, as well as the rationale and the limitations of ECMO. The benefit and the drawbacks associated with ECMO use along with a direct comparison with less invasive therapeutic strategies will be analyzed.

## Introduction

Mechanical ventilation (MV) constitutes one of the most researched and evolving areas of intensive care. In no other era has MV been better understood and safer applied than in the current decade. Despite the substantial progress, the harmful effects of MV cannot be erased and in the most difficult to ventilate patients, veno-venous extracorporeal membrane oxygenation (ECMO) is a promising intervention.

ECMO provides gas exchange via an extracorporeal circuit. The idea is not new; from 1966 to 1979, ECMO was used in several hundreds of patients with acute respiratory failure [1, 2]. The technology was rather crude at that time and the first randomized trial by Zapol et al. demonstrated no benefit at all, along with major complications, mostly bleeding [3]. These results sidelined the use of ECMO for respiratory failure for several years. It continued to be applied mostly in severe hypoxemia in neonates since the mid-1970s [4]. A renewed impetus in ECMO use for acute respiratory failure arose with the H1N1 flu epidemic in 2008–2009 followed by two randomized clinical trials. Since then, interest in ECMO has risen exponentially.

There are various configurations of ECMO, depending on whether the aim is to support the lungs, the heart, or both. In this narrative review, we will focus on the use of veno-venous ECMO (v-v ECMO) for acute hypoxemic respiratory failure. This review attempts to describe together the practical approaches that can be offered to the sickest patients before going to ECMO, as well as the rationale and the limitations of ECMO. This corresponds to the needs of clinicians taking care of these patients.

## Veno-venous ECMO: technique and physiology of gas exchange

**Fig. 1:**
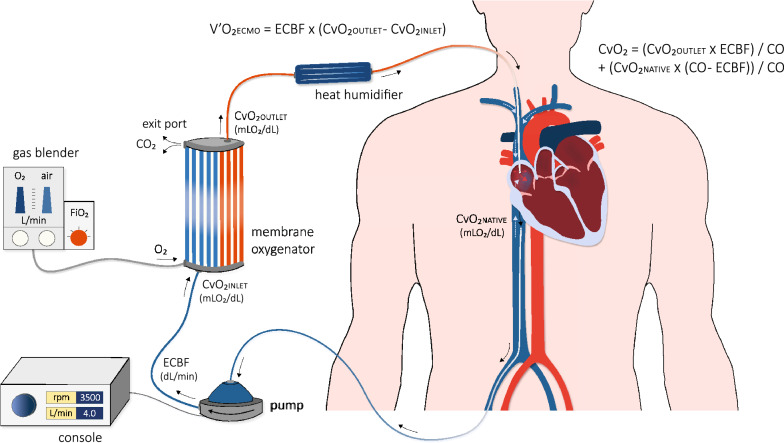
Illustration of the ECMO technique and physiology of gas exchange (see main text for a detailed description). Venous blood is drawn via a central venous catheter inserted in the femoral vein, which is propelled at a set extracorporeal blood flow (ECBF) rate to a membrane oxygenator where gas exchange takes place. Gas with a user-adjusted fraction of O_2_ enters the oxygenator in order to saturate the hemoglobin of the venous blood. CO_2_ diffuses from the venous blood to the gas and leaves the oxygenator via an exit port. Heated and humidified oxygenated blood is then returned to the venous circulation via the jugular vein. V’O2 ECMO reflects the amount of oxygen delivered by the ECMO system and is depending on the ECBF and the oxygen content in the blood before (CvO_2INLET_) and after (CvO_2OUTLET_) the membrane oxygenator, respectively. White arrows in the subclavian veins and vena cava indicate native venous return; the oxygen content of the native system (CvO_2NATIVE_) is mixed with the oxygenated blood from the ECMO system. The arterial oxygen content of the mixed blood (CvO_2_) is described by Equation 2 and depends on the ratio of ECBF to cardiac output (CO_2_). CO, cardiac output; CO_2_, carbon dioxide; CvO_2_, oxygen content in venous blood; ECBF, extracorporeal blood flow; O_2_, oxygen

Figure 1 illustrates the working principle of v-v ECMO. ECMO draws blood from the venous system, enriches it with oxygen, removes carbon dioxide (CO_2_), and returns the final product again to the venous circulation. Blood is withdrawn via a central venous catheter and is subsequently propelled to a membrane oxygenator [5]. Gas with a certain amount of inspired fraction of oxygen (FiO_2_) is pumped into the membrane oxygenator, where gas exchange takes place: CO_2_ diffuses from the venous blood to the gas, while oxygen leaves the gas to saturate the hemoglobin (Hb) of the venous blood. CO_2_ is continuously removed from the membrane oxygenator through an exit port [5]. Haldane and Bohr effects play a significant role in this process.

### Oxygenation

While arterio-venous ECMO improves both arterial oxygen content (CaO_2_) and cardiac output (CO), v-v ECMO affects only CaO_2_. The amount of oxygen delivered by ECMO to the extracorporeal blood (V′O_2ECMO_, ml/min) is described by the following equation:

1 $${\text{V}}^{'} {\text{O}}_{{{\text{2ECMO}}}} = {\text{ECBF}} \times \left( {{\text{CvO}}_{{{\text{2outlet}}}} - {\text{CvO}}_{{{\text{2inlet}}}} } \right),$$

where ECBF is extracorporeal blood flow in dl/min and CvO_2inlet_ and CvO_2outlet_ are the oxygen content in the venous blood before and after the membrane oxygenator, respectively, (ml O_2_/dl of whole blood) [5].

The ECBF, externally regulated on the ECMO module interface, is the most significant factor affecting oxygenation in ECMO. Resistance to blood flow can diminish the maximum ECBF that can be achieved and is related to catheter characteristics (length, diameter, site of the drainage), cardiac output, and membrane oxygenator function. The CaO_2outlet_ can be increased by increasing FiO_2_ in the sweep gas or by raising the Hb.

When two systems, ECMO and native venous return, provide blood of different concentrations of O_2_ at different flows rates, the arterial oxygen content in the mixed blood (CvO_2_) is the average of the amount of oxygen in each of the two systems [6]:

2 $$\text{CvO}_2 = \frac{\text{CvO}_2 \,\text{outlet} \,\times\,\text{ECBF}}{\text{CO}} + \frac{\text{CvO}_2\, \text{native}\, \times \,\left( \text{CO} - \text{ECBF} \right)}{\text{CO}},$$

where CO indicates cardiac output and CvO_2native_ the oxygen content of the native venous return. Equation (2) dictates that the ECBF/CO ratio is crucial: the higher the ratio, the higher the C$v$O_2_ and the oxygen saturation (SvO_2_) and partial pressure of O_2_ in the mixed venous blood entering the pulmonary artery. For ECBF to be sufficiently high to correct severe hypoxemia, large cannulas are required. Two factors could limit the efficiency of gas exchange correction. Firstly, high C$v$O_2_ and SvO_2_ in the pulmonary circulation offered by ECMO globally tend to increase oxygenation, but paradoxically reduce or eliminate hypoxic pulmonary vasoconstriction, which increases pulmonary shunt [7]. The vasoconstriction of pulmonary vessels in the presence of hypoxia, constitutes an important protective physiological reflex, that improves ventilation–perfusion and shunt by redirecting blood flow away from hypoxic areas of the lungs to better ventilated and oxygenated regions [8]. This is why there is a kind of competition between the oxygenated blood flow brought by ECMO and the native blood flow. This explains why ECMO blood flow needs to be very high compared to native blood flow. Secondly, a fraction of oxygenated blood from ECMO re-enters the membrane oxygenator without prior mixing with systemic circulation (recirculation) and may contribute to hypoxemia [9]. Both factors explain why increasing oxygen delivery from ECMO has less than predicted impact on PaO_2_ and SaO_2_.

### CO_2_ removal during ECMO

CO_2_ removal during ECMO depends on the sweep gas flow rate and the ECMO blood flow rate. Increases in sweep gas flow rate results in faster CO_2_ clearance from the oxygenator and thus higher CO_2_ removal from the blood. As CO_2_ is more soluble and diffusible in blood than O_2_, decarboxylation is achieved faster than oxygenation for the same blood flow rate. Hence, oxygenation in ECMO mostly depends on the blood flow rate while decarboxylation is primarily regulated by the sweep gas flow rate. Notably, oxygenation and CO_2_ removal through ECMO are interrelated: CO_2_ removal and consequent alkalinization of blood enhances the oxygen uptake (Bohr effect), while oxygenation promotes CO_2_ removal from blood as oxygenated hemoglobin has decreased capacity to carry CO_2_ (Haldane effect) [10].

## Indications and contraindications

The principal indications for v-v ECMO are acute, refractory hypoxemia, usually in the context of acute respiratory distress syndrome (ARDS) [9, 11–16]. In this scenario, v-v ECMO is applied whenever MV fails to ensure adequate oxygenation and/or when lung protective ventilation, with reasonable plateau and driving pressures is not feasible. Prone position should be offered first before considering ECMO. v-v ECMO is also used as a bridge to lung transplantation or after primary graft dysfunction following lung transplantation [17, 18]. It is contraindicated in patients with irreversible underlying lung disease who are not candidates for lung transplantation. Moreover, its use is discouraged in conditions precluding recovery or unacceptable functional outcome if the patient recovers (i.e., moribund state, metastatic malignancy, hypoxic brain injury, etc.).

## Evidence of benefit from veno-venous ECMO

The modern ECMO machines have little to do with those used in late 1970s. Improvements in the efficacy, ease of use, and, more importantly, safety, accelerated the widespread use of ECMO during the H1N1-associated ARDS in 2009. In that same year, the results of the first major randomized controlled trial employing v-v ECMO for severe ARDS were published [15]. In the CESAR trial, 180 adults with severe ARDS were randomized to be either transferred to one single center with ECMO capability or to remain at any of the 68 hospitals in the UK where they were already receiving conventional management [15]. The primary composite endpoint, death or severe disability at six months, was significantly lower for patients randomized to receiving ECMO (37% vs 53%, *p* = 0.03), with an absolute risk reduction of 16%. In reality, the CESAR trial did not directly compare ECMO to no ECMO; 24% of patients in the ECMO group never received ECMO. Almost all of them (93%), however, underwent protocolized lung protective MV. Contrariwise, a lung protective ventilation strategy was not mandated for patients in the control group and only 70% did, at some point during their course, receive low-volume, low-pressure MV. Prone positioning was employed in only 42% of control and 37% of ECMO patients [19]. Hence, the CESAR trial did not conclusively prove that ECMO was effective, but did show the importance of lung protective specialized management in severe ARDS.

Recently, an international randomized controlled trial [ECMO to rescue Lung Injury in severe ARDS (EOLIA trial)] compared early v-v ECMO with standard lung protective ventilation in patients with severe ARDS [12]. Patients with severe ARDS receiving MV for less than 7 days were randomized to standard of care, including protocolized MV (*n* = 125) or to early ECMO (*n* = 124). The entry criteria were severe hypoxemia (PaO_2_/FiO_2_ < 50 or 80 mmHg for > 3 or > 6 h, respectively) or hypercapnia (PaCO_2_ ≥ 60 mmHg with a pH < 7.25 for > 6 h with the respiratory rate increased to 35 breaths/minute and ventilation settings adjusted to keep a plateau pressure of ≤ 32 cmH_2_O) despite ventilator optimization. The later included an FiO_2_ of  ≥ 0.80, a tidal volume of 6 ml per kilogram of predicted body weight (PBW), and a positive end-expiratory pressure (PEEP) of  ≥ 10 cmH_2_O. Neuromuscular blocking agents and prone position were strongly encouraged. Crossover from control to ECMO for refractory hypoxemia was allowed if the patients had refractory hypoxemia despite the use of available and feasible adjunctive therapies. Ultra-protective ventilation was applied to patients on ECMO with further tidal volume and respiratory rate reduction. The study failed to achieve the goal of 20% reduction in 60-day mortality and was terminated early for futility. Although not statistically significant, the 11% absolute reduction in mortality with ECMO was considered by many physicians as clinically important. Therefore, the results of the EOLIA trial were re-examined through the prism of Bayesian analysis, which combines a prior probability function (calculated from prior data and beliefs), with a likelihood function (calculated from new data) to create a posterior probability function [20, 21]. The latter is an updated summary of knowledge and the remaining uncertainty. Bayesian analysis of the EOLIA trial demonstrated that the posterior probability of any reduction in mortality with ECMO was very high (88–99%) and the probability of an absolute risk reduction ≥ 2% ranged from 78 to 98% [20].

Pooled mortality data from the CESAR and EOLIA trials and from three observational studies on v-v ECMO for severe ARDS (total of 773 patients), in which matching techniques were used, disclosed that ECMO was associated with a significant reduction in 60-day mortality compared with conventional ventilation (relative risk, 0.69; 95% CI 0.50–0.95) [22]. Nevertheless, there was a moderate risk of major 7hemorrhage and a low risk of cannula- and circuit-related complications.

The reported use of ECMO in recent ARDS trials is presented in Table 1.

**Table 1:** Reported use of ECMO in recent ARDS studies

Study	Intervention	Population	Total patients N	Total ECMO use n (% of N)	ECMO use in intervention group n (% of N)
ACURASYS [95]	Cisatracurium	Severe ARDS (PF ratio < 150 mmHg)	339	0 (0)	0 (0)
ART [120]	Lung recruitment maneuvers	Moderate-to-severe ARDS (PF ratio ≤ 200 mmHg)	1010	10 (0.99)	5 (0.5)
EPVENT-2 [121]	Transpulmonary pressure targets	Moderate-to-severe ARDS (PF ratio ≤ 200 mmHg)	200	4 (2.0)	1 (0.5)
ICU-ROX [122]	Conservative oxygen	PF ratio < 300 mmHg	623	13^a^ (2.09)	7^a^ (1.12)
LIVE [123]	Personalized mechanical ventilation	Moderate-to-severe ARDS (PF ratio ≤ 200 mmHg)	400	14 (3.5)	7 (1.75)
LOCO_2_ [124]	Liberal oxygen	Mild-to-severe ARDS (PF ratio ≤ 300 mmHg)	201	0 (0)	0 (0)
LUNG SAFE [73]	NA	Mild-to-severe ARDS (PF ratio ≤ 300 mmHg)	2377	76 (3.2)	N/A
PHARLAP [125]	Lung recruitment maneuvers	Moderate-to-severe ARDS (PF ratio ≤ 200 mmHg)	114	7 (6.14)	1 (0.88)
PROSEVA [68]	Prone positioning	Severe ARDS (PF < 150 mmHg)	466	8 (1.72)	2 (0.43)
ROSE [69]	Cisatracurium	Moderate-to-severe ARDS (PF ≤ 200 mmHg)	1006	13 (1.29)	3 (0.30)
Total			6736	145 (2.15)	

^a^The number of patients reported (N) reflects the use of extracorporeal membrane oxygenation (ECMO) and extracorporeal carbon dioxide removal (ECCO_2_R). NA non-applicable; ARDS, acute respiratory distress syndrome; PF,  ratio of partial pressure of oxygen to inspired fraction of oxygen

## ECMO use in pandemics

During the global pandemic of influenza H1N1 in 2009–2010, collaborative networks from Australia and New Zealand (ANZICS), France (the REVA Network), Italy and the UK reported survival rates of 70% in patients with severe ARDS treated with ECMO [11, 13, 14, 16]. At the same time, however, other centers published equally auspicious outcomes without the use of ECMO [23]. Matched score analysis from the UK and REVA network presented inconsistent results in terms of mortality [13, 16].

The new pandemic of coronavirus disease 2019 (COVID-19) is complicated by ARDS at rates ranging from 5 to 42% [24, 25]. In the first retrospective studies of patients with severe COVID-19 who received ECMO, mortality was sometimes as high as 80–94% [24–35]. Mortality was noticeably lower (31–54%) in subsequent multicenter studies, conducted in younger patients [36–40]. In critically ill patients with COVID-19, it has been recently shown that presenting ICU physiology, but also hospital socioeconomic status, hospital capacity and strain contribute to significant interhospital variation in observed mortality [41]. This finding may also explain why studies report different outcomes for ECMO in COVID-19, especially patient factors. The first nationwide study, coming from Chile, demonstrated that, during the 1st wave of the pandemic, ECMO was employed in 14.89:100,000 patients with COVID-19 representing 0.42:100,000 population, and 1.2% of COVID-19 intubated patients) [42]. The 90-day mortality of ECMO supported COVID-19 patients was 38.8%, comparable to that reported for other indications of ECMO. Lower respiratory system compliance and higher driving pressure before ECMO were associated with higher mortality. Outcomes of studies investigating v-v ECMO in severe COVID-19 are presented in Table 2.

**Table 2:** Studies reporting outcomes of veno-venous ECMO in COVID-19 critically ill adults

Study	Study type	Age	Pre-ECMO MV (days)	ECMO usage	Pre-ECMO PaO_2_/FiO_2_	Prone before ECMO	NMB before ECMO	ECMO duration (days)	Mortality
Yang [34]	Retrospective/single center	NA	NA	6/52	NA	NA	NA	NA	83.3%
Osho [31]	Case series	47 (43–54)	5.5 (3.5–6.75)	6/6	95 (84–100)	100%	100%	12 (4–18)	8%^a^
Li [29]	Retrospective/multicenter	64.3 (± 17.6)	9.7 (± 5.7)	8/16	66.1 (± 7.8)	NA	NA	27.1 (± 17.7)	50%^a^
Zhang [126]	Retrospective/single center	NA	NA	10/221	NA	NA	NA	NA	20%^a^
Zeng [35]	Retrospective/2 centers	50.9 (13.5)	NA	12/12	NA	NA	NA	11.3 (± 7.8)	42%^a^
Le Breton [127]	Case series	50 (43–55)	6 (4–6)	13/83	59 (54–58)	100%	100%	13 (3–34)	85%
Yang [34]	Retrospective/multicenter	62 (33–78)	4 (1–7)	73/73	71.9 (59–87)	58.9%	NA	18.5 (12–30)	81%^a^
Jacobs [27]	Retrospective/multicenter	52.4 (± 12.5)	4 (2.0–6.5)	32/32	NA	62.5%	NA	6 (5–10)	31%^a^
Lebreton [39]	Retrospective/multicenter	52 (45–58)	5 (3–7)	302/302	61 (54–70)	94%	96%	14 (8–26)	54%
Kon [28]	Retrospective/single center	40 (30.5–47)	2 (1–4)	27/321	84 (70–118)	82%	96%	11 (10–14)	4%^a^
Jacobs [37]	Prospective, multicenter cohort	51 (41–60)	4 (1–6)	100/100	NA	70%	NA	12 (8–22)	50%
Schmidt [40]	Retrospective/multicenter	49 (41–56)	4 (3–6)	83/492	60 (54–68)	94%	96%	20 (10–40)	31%
Barbaro [36]	Retrospective/multicenter	49 (41–57)	4 (1.8–6.4)	1035/1035	72 (59–64)	60%	72%	14 (8–23)	39%
Mustafa [30]	Retrospective/two centers	48.4 (± 1.5)	4 (± 0.5)	40/40^b^	NA	73%	78%	13 (± 2.6)	15%
Diaz [42]	Retrospective/multicenter	48 (41–55)	4 (2–7)	85/85	87 (64–99)	91.8%	94.1%	16 (10–27)	38.8%

Data are presented as mean (± standard deviation) or median (interquartile range). ECMO extracorporeal membrane oxygenation, MV mechanical ventilation, NMB neuromuscular blocking agents, NA non-applicable

^a^Treatment continues for many patients and there was no final outcome at the time of publication

^b^Refers to veno-venous ECMO with single-access, dual-stage right atrium-to-pulmonary artery cannula implantation

Recently, protection of the right heart with single-access, dual stage (right atrium-to-pulmonary artery cannulation) v-v ECMO or ECMO with right ventricular assist device yielded promising results in severe COVID-19 disease, but further studies are required to assess the value of this approach [30, 43].

## Complications

Even with modern ECMO circuits, complications associated with the technique are manifold, can be devastating, and their incidence is not precisely known and probably underestimated, since they are not consistently reported across studies. These complications are broadly divided into mechanical (i.e., associated with device insertion or function) and medical (i.e., associated with either anticoagulation or the effect of ECMO on human organism) complications (Table 3).

**Table 3:** ECMO complications

Category	Event	Mechanism
Mechanical	ECMO malfunction	Tubing kinking
	Oxygenator failure	Tubing rupture/disconnection/blood clots Blood clots or air in circuit or oxygenator
Insertion-related	Vascular bleeding	Vessel injury
	Vascular dissection	
	Vascular thrombosis	
	Air embolism	Air in blood
	Limb ischemia	Vessel injury
	Pneumothorax	Organ injury
	Organ perforation	
Medical		
Hematologic	Non-traumatic bleeding	Anticoagulation
	Thrombosis/embolism	Platelet dysfunction or consumption
	Hemolysis	Hemostatic derangement
Neurologic	CSN bleeding	Bleeding
	CNS ischemia	Thrombosis/hypotension
	CNS infarction	Loss of cerebral autoregulation
	Seizures	Arterial pressure variations PaCO2 changes Reperfusion injury
Inflammatory	Infections/SIRS	Microorganisms/inflammatory activation
Renal	Acute kidney injury	Renal hypoperfusion Ischemia–reperfusion Fluid overload SIRS/sepsis Coagulopathy

CNS, central nervous system; SIRS, systemic inflammatory response syndrome

### Device-related complications

The commonest adverse events reported are bleeding at the site of cannulation (7.8%), vascular injury, insertion site infection and deep venous thrombosis (DVT). Cannulation of improper vessels, retroperitoneal injury, limb ischemia, air embolism, and, rarely, cardiac perforation during guidewire advancement may occur.

Circuit failure occurs in 13–25% of cases, most often due to clot formation within the circuit or oxygenator. Other reasons include oxygenator failure (5.9%), gas in the circuit and pump failure. The most dreadful circuit-related complications are massive gas embolism, resulting from extremely negative pressures between the drainage cannula and ECMO pump, and massive blood loss secondary to tubing disconnection or breach. ECMO necessitated exchange in 31% of 265 ARDS patients and in 45% of these cases the exchange was required on an urgent basis [44].

### Medical complications

#### Bleeding and thrombosis

The exposure of blood to non-endothelial surfaces and high shear stress activates the adhesion of platelets, thrombin generation and clot formation in the cannula or circuit. Furthermore, the coagulation cascade is triggered by endothelial injury from intravascular catheters and blood flow disturbances. The true incidence of thrombosis is unknown. When systematically investigated, cannula associated DVT develops in 62–85% of patients undergoing v-v ECMO and with a 16% incidence of pulmonary embolism [45–47]. Systemic anticoagulation does not abolish the risk of thromboembolism.

Hemorrhage complicates the course of 16–50% of patients receiving v-v ECMO [48–50] with the commonest sites being the cannula site (13.2%), gastrointestinal tract (5.5%), lungs (6.1%) and central nervous system (3.9%) [48]. Hemorrhage is one of the leading causes of morbidity and mortality in patients undergoing ECMO [48, 51]. Ried et al. demonstrated a bleeding rate of 23.2% in v-v ECMO, with a mortality rate of 48.5% among these patients [51]. Very high rates of major bleeding requiring transfusion (43%) complicated the application of ECMO in a recently published large multicenter study of COVID-19 patients [39]. The etiology of ECMO-related bleeding is multifactorial and it is related to the circuit, the systemic anticoagulation and the patient. Thrombocytopenia has a considerable prevalence (up to 21%), regardless of the type of ECMO mode, and is commonly complicated with bleeding [50].

#### Hemolysis

Intravascular hemolysis occurs commonly under ECMO (5–18%) [52]. It is caused by shear stress and erythrocyte breakdown resulting from turbulent flow either within the circuit, due to partial obstruction from the thrombus, fibrin deposition in the pump and/or negative pressure in the access line or, less frequently, in the systemic circulation due to intravascular thrombosis. Released extracellular Hb causes vasoconstriction, endothelial dysfunction and platelet aggregation. Hemolysis is especially frequent at lower blood flow rates, such as those used for extracorporeal CO_2_ removal [53].

#### Neurologic complications

Approximately 7–9% of patients suffer from neurologic injury during v-v ECMO [54, 55].The clinical spectrum ranges from neurocognitive disturbances to seizures (1.1%), ischemic stroke (1.9%), intracranial hemorrhage (3.5%), and brain death (1.6%) [54]. The pathophysiology includes hypotension (cardiac arrest, shock, etc.), loss of cerebral autoregulation, large arterial pressure variations during ECMO, reperfusion injury, anticoagulation, and embolism. Brain hemorrhage and ischemic stroke are the most devastating neurologic complications associated with a mortality rate of 60–96% and 50–84%, respectively, and significant long-term functional impairment [56]. Intracerebral hemorrhage was reported as the commonest cause of death in H1N1 influenza patients treated with v-v ECMO [11, 57]. Intracerebral hemorrhage occurred in 12–13% of patients with COVID-19 treated with ECMO [39, 42], and the incidence was higher than that reported for ECMO for ARDS caused by other viruses [58]. It is worth mentioning that the EOLIA study was the only one that did not find an increase in intracranial bleeding with ECMO, probably reflecting that experience in ECMO use is associated with safer use of anticoagulation [12]. Importantly, sudden changes in PaCO_2_ have been independently associated with neurological complications [54, 57, 59]. In a large international study, a PaCO_2_ decrease greater than 50% in the first 24 h following ECMO onset significantly increased all neurologic complications [54].

#### Sepsis and systemic inflammatory syndrome

In all extracorporeal therapies, the exposure of blood to non-biological surfaces, the blood shear stress and the air–blood interface initiate and amplify a systemic inflammatory response. The inflammatory response to ECMO is intricate and involves the triggering of a variety of coagulation and inflammatory cascades. Widespread endothelial injury, capillary leaks and multiple organ dysfunction is possible [60, 61]. More than 50% of patients develop an infection while on ECMO therapy, predominantly ventilator-associated pneumonia (35%), and 26% develop sepsis with significant increase in mortality [52, 62].

#### Renal failure

More than two-thirds of patients develop acute kidney injury during ECMO treatment and approximately 50–65% of them eventually need renal replacement therapy [52, 63]. Beyond patient-related factors, ECMO may harm the kidneys through multiple mechanisms: (1) renal hypoperfusion in case of bleeding, hemolysis, catheter obstruction and vascular thromboembolism; (2) fluid overload; (3) ischemia–reperfusion injury promoted by hemodynamic fluctuations and renin–angiotensin–aldosterone dysregulation; (4) systemic inflammatory response syndrome, and (5) coagulopathy and catheter-related infections.

## ECMO versus conventional management

**Fig. 2:**
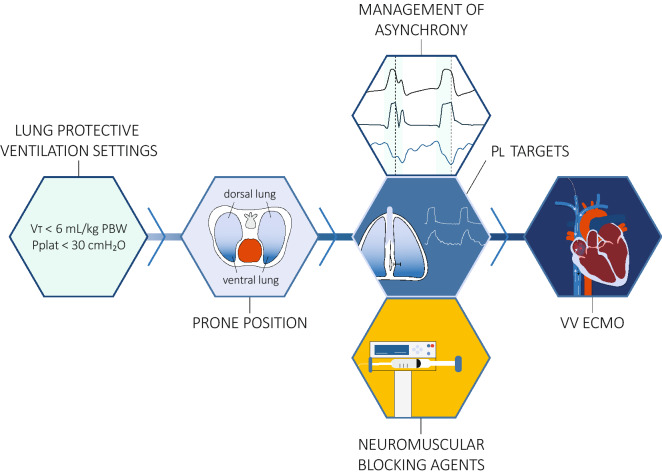
Illustration of therapeutic options in ARDS patients that should be considered prior to employing ECMO treatment. Equal weight is given to the management of asynchrony, transpulmonary pressure (PL) targets, and administration of neuromuscular blocking agents, in order to illustrate that specific patients may benefit from specific strategies (e.g. PL measurements in obese patients, and paralysis in patients with high respiratory drive or reverse triggering (entrainment) despite sedation). VT, tidal volume; Pplat, plateau pressure; VV ECMO, veno-venous extracorporeal membrane oxygenation

Prior to employing a complex, costly and potentially dangerous treatment such as ECMO, the clinician should exhaust any available simpler, less resource intensive and safer therapeutic options (Fig. 2). To date, there are two conventional interventions with proven survival benefit in ARDS patients, namely lung protective ventilation with low tidal volumes (6 ml/kg PBW) and prone position [64–70]. Resolution of patient–ventilator dyssynchronies, application of sufficient PEEP, administration of neuromuscular blocking agents, and transpulmonary pressure measurements constitute further strategies that may benefit patients.

### Lung protective ventilation

The application of “gentle” mechanical ventilation with low tidal volumes and plateau and driving pressures, low respiratory rates and low mechanical power has been shown to reduce the risk and magnitude of VILI [66, 67, 70–72]. Before proceeding to ECMO for lung protection, the abilities of conventional ventilation should be fully exploited. Remarkably, the LUNG SAFE study demonstrated that the principles of lung protective ventilation were not rigorously respected in clinical practice: plateau pressure was measured in only 40% of patients with ARDS, whereas only two-thirds of patients in whom respiratory system mechanics were calculated actually received lung protective ventilation [73]. Furthermore, the inverse relationship between FiO_2_ and PaO_2_ and the absence of correlation between PEEP and PaO_2_/FiO_2_ indicated that physicians primarily used FiO_2_ to treat hypoxemia. Thus, even following ECMO initiation, many patients continue to have injurious ventilator settings.

The employment of ultra-protective ventilation is appealing in patients with severe ARDS. The LIFEGARDS international study showed that ECMO permitted ultra-lung protective ventilation: tidal volume and plateau pressures were both significantly reduced from 6.4 ± 2.0 to 3.7 ± 2.0 ml/kg PBW and from 32 ± 7 to 24 ± 7 cmH_2_O, respectively [74]. Notwithstanding, ultra-low tidal volume ventilation can also be applied in approximately two-thirds of moderately severe to severe ARDS patients without extracorporeal treatment [75], while it is not without risks: it can lead to lung collapse with associated hypoxemia and compliance deterioration and is frequently accompanied by severe acidosis [75, 76]. Finally, whether very-low tidal volume ventilation with ECMO is superior to conventional low ventilation is largely unknown.

### Recruitment and positive end-expiratory pressure

Applying appropriately sufficiently high end-expiratory pressure (PEEP) is an essential component of ARDS ventilation. Adequate PEEP recruits atelectatic regions, improves regional lung compliance, decreases shunt, and reduces cyclic closing and reopening of alveoli without increasing stress and hemodynamic compromise [77]. Although, higher versus moderate PEEP for the same degree of hypoxemia had not shown survival benefit in the general population with ARDS [78–80], subsequent individual patient-data meta-analysis, indicated that higher PEEP was associated with lower mortality in hypoxemic patients with PaO2/FiO2 < 200 mmHg and with no benefit in mild ARDS [81].

### Prone position

Prone position improves oxygenation, reduces the risk of VILI and may improve right heart function in patients with hypoxemic respiratory failure. The redistribution of lung densities with a recruitment of dorsal regions associated with increase in chest wall elastance, follow a more homogenous ventilation distribution, while alveolar shunt reduction results from improvement in ventilation/perfusion matching [82]. Gattinoni et al. demonstrated that prone position significantly improved arterial oxygenation in ARDS patients and might have a survival advantage in those with very severe hypoxemia[69]. The landmark PROSEVA trial by Guerin et al. proved that long-term prone positioning significantly reduced 28-day mortality (16% vs 32.8%, *p* < 0.001) in ARDS patients with PaO_2_/FiO_2_ < 150 mmHg [68]. Meta-analyses confirmed that prone position carries a substantial survival benefit, provided that it is implemented in severely hypoxemic patients and for longer than 12-h sessions [83–85]. Complications are infrequent and include tube dislodgement or obstruction, pressure ulceration and, rarely, cardiac arrest. For sustained oxygenation improvement, prone position sessions should last and should be repeated several times. In the PROSEVA study, patients underwent four prone position sessions on average, which lasted 17 consecutive hours each, even if oxygenation did not improve following proning [68]. This recommendation emphasizes that the beneficial effect of prone position in patients with severe hypoxemia is expected mainly through the increase in lung homogeneity and the associated reduction of regional stress and strain [86] and, secondarily, through improvement in oxygenation [87]. Therefore, beyond PaO_2_/FiO_2_ changes following proning, reduction of plateau airway pressure, driving pressure and transpulmonary pressure (if available) should be evaluated as well. The optimal duration of prone position is not known. In patients with COVID-19 related lung injury, proning sessions of 36 h could be safely performed and were associated with more sustained oxygenation improvement compared to standard 16 h of proning [88].

Given the evidence, one would expect that clinicians would enthusiastically embrace a simple, relatively safe intervention, with proven survival benefit and no additional cost. Nevertheless, data from subsequent international studies indicate a radically different clinical attitude. In the LUNG SAFE study, proning was used in only 16.3% of patients with severe ARDS [73]. Similarly, Li X et al. disclosed that not more than 31% out of 672 ARDS patients managed with v-v ECMO had previously underwent a trial of prone positioning [89]. Remarkably, the positive results of Guerin et al. [68] failed to change routine clinical practice at least before the COVID-19 pandemic. The proportion of patients in whom prone positioning was used before ECMO was lower in the more recent studies (19%) versus those published before 2013 [89]. Even in experienced ECMO centers, prone position was offered to only 26% of patients [74]. In a recent multinational survey, only 21% of experienced v-v ECMO physicians would be reluctant to initiate ECMO without beforehand turning patients prone [90]. In a prospective international 1-day prevalence study, the rate of proning was higher in non-European than in European countries (28.6 vs 13%; *p* = 0.019) [91]. Notably, prone position is part of pre-ECMO support in no more than 60% of patients with COVID-19 (https://www.elso.org/Registry/FullCOVID19RegistryDashboard.aspx).

### Neuromuscular blocking agents

Spontaneous breathing activity, under the influence of high respiratory drive with or without MV, may harm the lungs due to patient–ventilator dyssynchrony, excessive transpulmonary and transmural vascular pressures and pendelluft phenomenon [92]. The higher the respiratory drive, the more injurious are the inspiratory efforts for the lung. Neuromuscular blocking agents abolish spontaneous breathing activity and they may have anti-inflammatory properties. The ACURASYS study and two meta-analyses demonstrated a reduction in mortality and days on MV and improved oxygenation in the most hypoxemic ARDS patients treated with continuous neuromuscular blockade [93–95]. The incidence of ICU acquired weakness did not increase as a result of cisatracurium. Subsequent studies did not find a survival benefit from early administration of neuromuscular blocking agents in ARDS [65, 96], but it still seems prudent to consider their use prior to ECMO in the sickest patients. This is not often the case in clinical practice, though: around 40% of patients received ECMO without any trial of neuromuscular blockade as reported in the LIFEGARDS study [74].

### Transpulmonary pressure targeted ventilation

Recruitment and ventilation settings adjusted to reach targets of transpulmonary pressures (*P*_L_), measured via an esophageal catheter, allow individualization of MV and have been shown to improve oxygenation while, concomitantly, reduce the risk of lung injury [97–100]. Transpulmonary pressures calculation allows the partitioning of respiratory mechanics between the lung and chest wall. In influenza A (H1N1)-associated ARDS patients referred to an ECMO center, setting PEEP to approach a maximum end-inspiratory PL of 25cmH2O improved oxygenation and obviated the use of ECMO for the group of patients with high chest wall elastance (50% in this study) [101]. Similarly, an end-expiratory *P*_L_ guided open lung approach significantly increased oxygenation and obviated the use of ECMO in all 8 patients with severe ARDS (PaO_2_/FiO_2_ 62 ± 7 mmHg) not responding to any conventional ventilation management, including neuromuscular blockade and prone positioning [102]. Individual titration of mechanical ventilation based, among others, on esophageal manometry might be particularly valuable in obese patients and could improve their outcome compared to standard practice [103]. In this group, the high prevalence of complete airway closure (> 41%), can make driving pressures unreliable [104]. Furthermore, the hemodynamic tolerance to high PEEP was found remarkably good in class III obese patients (mean BMI = 57 kg/m^2^) [105]. The technique is largely neglected in daily practice, likely due to technical challenges.

## Ethical considerations

From an ethical perspective, the decision to apply ECMO can be complex [106]. There are clinical scenarios in which the technique is clearly futile [90], but, in most cases it is noticeably difficult to predict outcome. Survival predicting tools have been proposed [107, 108], but the personal perception of “futility” and patient´s wishes and values, often expressed by family, play an important role [90]. Judgement may be clouded by emotional and physical stress, the hope offered by sophisticated therapies, or the inputs from friends or media. Additional limitations further challenge the decision to initiate ECMO during pandemics [109]. Even more intricate is to determine when to withdraw the treatment. All these aspects cannot be addressed by algorithms or guidelines. A thorough and honest discussion between the physician and the patient or his relatives regarding the benefits, risks and expectations of the treatment is essential. The goal should be the bridge to recovery [106, 110, 111].

## Summary and critical appraisal

Extracorporeal membrane oxygenation is, undoubtedly, a powerful aid against refractory hypoxemia and the inability to deliver lung protective ventilation with MV alone. It is also a highly invasive technique with many serious complications. Moreover, resource implications associated with ECMO are substantial: according to guidelines, ECMO should be employed in centers with experienced and organized multidisciplinary ECMO teams, available 24 h per day, with high nurse-to-patient ratio and where blood bank, radiology and vascular, thoracic and abdominal surgical departments are directly disposable to address emergencies [112]. Furthermore, it is definitely an expensive modality: financial analyses estimated that ECMO raised the costs per patient by approximately 57.000 to 70.000 USD [15, 113] or at 30.000 USD/quality-adjusted life year [114]. Concurrently, existing evidence suggests that v-v ECMO might have a mortality benefit in a very small portion of patients: those with very severe hypoxemia (PaO_2_/FiO_2_ < 80 mmHg), who receive injurious mechanical ventilation settings and in whom all conservative strategies such as lung protective ventilation, repeated prone positioning and neuromuscular blocking agents have failed [115]. Table 2 details the reported use of ECMO as adjunctive therapy in recent large randomized clinical trials in a total of 6736 ARDS patients; only 2.15% of patients required ECMO, which ranged from 0 to 6.14% for individual studies. Finally, there is still no confirmatory data that ECMO, even in severely hypoxemic patients, prevails over optimal conventional management techniques.

Despite the above considerations, ECMO use exploded in the last decade and continues to rapidly expand worldwide, even in small hospitals. Internationally, between 2010 and 2019, the number of centers that provide ECMO at any age and for any cause increased from 183 to 4637 and the number of ECMO runs per year increased from 3445 to 12,850 according to the Extracorporeal Life Support Organization registry database [116]. Analyzing data from the Federal Statistical Office of Germany, Karagiannidis et al. found that v-v ECMO use almost tripled between 2007 and 2014, whilst mortality among patients who had received ECMO for less than 48 h was 70% [117]. This mortality rate was higher than that reported in epidemiologic studies on severe ARDS [73], indicating that either some patients were too sick to benefit from ECMO anyway and/or that dreadful complications affected the outcome [118].There is no definite answer why doctors resort so easily to a complicated treatment such as ECMO. Some physicians may perceive that ECMO implementation is more straightforward and, hence, less time consuming and less prone to errors as compared to the individualization of mechanical ventilation settings based on lung mechanics measurements. Nonetheless, proper use prerequisites a thorough understanding of the patient’s physiology. Other reasons include the belief that the more advanced and sophisticated the treatment, the higher the benefit for the patient, along with the excitement of dealing with advanced technology. Finally, financial interests should not be belittled: reimbursement from ECMO programs can be substantial for institutions and may constitute a strong incentive for its implementation [119].

## Conclusion

Lung protective ventilation, prone position, neuromuscular blockade and individualized ventilation driven by transpulmonary pressures, are efficient in the majority of hypoxemic patients, even in those with severe hypoxemia. v-v ECMO is a valuable tool in highly selected patients, in whom the aforementioned strategies fail to correct life threatening hypoxemia. This modality should be reserved after implementing simpler and less invasive strategies. Failure to do so may be harmful both for the patient as well as for the ECMO technique itself.

## Abbreviations

ARDS: Acute respiratory distress syndrome
ACURASYS: ARDS et Curarisation Systematique
CaO_2_: Arterial oxygen content
CvO_2_: Arterial oxygen content in the mixed blood
SaO_2_: Arterial oxygen saturation
CO_2_: Carbon dioxide
CO: Cardiac output
CESAR (study): Efficacy and economic assessment of conventional ventilatory support versus extracorporeal membrane oxygenation for severe adult respiratory failure [15]
EOLIA (study): ECMO to rescue Lung Injury in severe ARDS [12]
ECBF: Extracorporeal blood flow
ECMO: Extracorporeal membrane oxygenation
FiO_2_: Inspired fraction of oxygen
Hb: Hemoglobin
LUNG SAFE (study): Large observational study to UNderstand the Global impact of Severe Acute respiratory FailurE [73]
O_2_: Oxygen
CvO_2native_: Oxygen content of the native venous return
CvO_2outlet_: Oxygen content in the venous blood after the membrane oxygenator
CvO_2inlet_: Oxygen content in the venous blood before the membrane oxygenator
V’O_2ECMO_: Oxygen delivery by extracorporeal membrane oxygenation
SvO_2_: Oxygen saturation in the pulmonary artery
MV: Mechanical ventilation
PO_2_: Partial pressure of oxygen
PEEP: Positive end-expiratory pressure
PROSEVA (study): Prone positioning in severe acute respiratory distress syndrome[68]
PaO_2_/FiO_2_: Ratio of partial pressure of oxygen to inspired fraction of oxygen
*P*_L_: Transpulmonary pressures
v-v ECMO: Veno-venous extracorporeal membrane oxygenation
VILI: Ventilator-induced lung injury
LIFEGARDS (study): VentiLatIon management oF patients with Extracorporeal membrane oxyGenation for Acute Respiratory Distress Syndrome [74]
